# P16 expression and recurrent cervical intraepithelial neoplasia after cryotherapy among women living with HIV

**DOI:** 10.3389/fmed.2023.1277480

**Published:** 2023-10-10

**Authors:** Daniel Maina, Michael H. Chung, Marleen Temmerman, Zahir Moloo, Jonathan Wawire, Sharon A. Greene, Elizabeth R. Unger, Nelly Mugo, Samah Sakr, Shahin Sayed, Christine J. McGrath

**Affiliations:** ^1^Department of Pathology and Laboratory Medicine, Aga Khan University Hospital, Nairobi, Kenya; ^2^Division of Infectious Diseases, Department of Medicine Emory University, Atlanta, GA, United States; ^3^Department of Global Health, University of Washington, Seattle, WA, United States; ^4^Centers for Disease Control and Prevention, Atlanta, GA, United States; ^5^Sexual Reproductive and Adolescent Child Health Research Program, Kenya Medical Research Institute, Nairobi, Kenya; ^6^Coptic Hospital, Nairobi, Kenya

**Keywords:** CIN2+, p16, recurrence, cryotherapy, HIV, cervical cancer, cervical lesions

## Abstract

**Background:**

The expression of p16 protein, a surrogate marker for high-risk human papillomavirus (hrHPV), is associated with cervical dysplasia. We evaluated correlates of p16 expression at treatment for high-grade cervical lesions and its utility in predicting the recurrence of cervical intraepithelial lesions grade 2 or higher (CIN2+) following cryotherapy among women with HIV.

**Methods:**

This is a subgroup analysis of women with HIV in Kenya with baseline cervical biopsy-confirmed CIN2+ who were randomized to receive cryotherapy and followed every six-months for two-years for biopsy-confirmed recurrence of CIN2+. P16 immunohistochemistry was performed on the baseline cervical biopsy with a positive result defined as strong abnormal nuclear expression in a continuous block segment of cells (at least 10–20 cells).

**Results:**

Among the 200 women with CIN2+ randomized to cryotherapy, 160 (80%) had a baseline cervical biopsy specimen available, of whom 94 (59%) were p16-positive. p16 expression at baseline was associated with presence of any one of 14 hrHPV genotypes [Odds Ratio (OR) = 3.2; 95% Confidence Interval (CI), 1.03–9.78], multiple lifetime sexual partners (OR = 1.6; 95% CI, 1.03–2.54) and detectable plasma HIV viral load (>1,000 copies/mL; OR = 1.43; 95% CI, 1.01–2.03). Longer antiretroviral therapy duration (≥2 years) at baseline had lower odds of p16 expression (OR = 0.46; 95% CI, 0.24–0.87) than <2 years of antiretroviral therapy. Fifty-one women had CIN2+ recurrence over 2-years, of whom 33 (65%) were p16-positive at baseline. p16 was not associated with CIN2+ recurrence (Hazard Ratio = 1.35; 95% CI, 0.76–2.40).

**Conclusion:**

In this population of women with HIV and CIN2+, 41% of lesions were p16 negative and baseline p16 expression did not predict recurrence of cervical neoplasia during two-year follow up.

## Introduction

Cervical cancer is the fourth leading cause of cancer-related deaths in women globally despite the availability of cervical screening procedures and effective treatment when detected early (1). Sub-Saharan Africa has the highest burden, accounting for 25% of the global mortality attributable to cervical cancer (2). In 2020, cervical cancer was the second most common cancer and the leading cause of cancer-related deaths among women in Kenya (1). Among women with HIV, higher incidence, persistence, and infections with multiple high-risk human papillomavirus (hrHPV) genotypes, as well as the degree of immunosuppression, contribute to an increased risk of cervical cancer compared to HIV-uninfected women (3, 4).

The increased risk of progression to cervical cancer in women living with HIV may be due to the destruction of CD4 cells by HIV and the resultant immunosuppression, leading to a higher likelihood of hrHPV genotypes establishing infection (5) and reactivation of lesions due to reduced clearance of hrHPV infections (6). A clinical trial among women living with HIV and cervical intraepithelial neoplasia grade 2 or 3 (CIN2/3) showed that persistence of hrHPV infection was associated with cervical disease recurrence in the two-years following treatment (7).

Cryotherapy is the standard treatment modality for most precancerous cervical lesions in low- and middle-income countries (LMICs) (8). It is a low-cost, relatively simple, effective and safe procedure which can be easily accessed in primary healthcare settings. In women with HIV, cryotherapy has been associated with lower risk of cervical HIV shedding compared to loop electrosurgical excision procedure (LEEP) (9). However, cryotherapy may be less effective for removing cervical lesions than other treatments such as LEEP, although evidence is limited and contradictory. A meta-analysis reported elevated risk of recurrence of cervical lesions in women living with HIV compared to HIV-uninfected women, yet showed no difference in recurrence of cervical disease among women with HIV treated with cryotherapy vs. LEEP (10). Conversely, a clinical trial among women with HIV in Kenya showed higher cervical intraepithelial neoplasia grade 2 or higher (CIN2+) recurrence following cryotherapy than LEEP (11).

Recurrence of CIN2+ disease after cryotherapy remains an important consideration in secondary prevention of cervical cancer, particularly in women with HIV who have an increased risk of persistent hrHPV infections and subsequent cervical disease. A biomarker that can predict a subset of women with HIV with elevated risk of CIN2+ recurrence could provide clinicians with a triage tool to help inform alternative treatment or the need for close monitoring following treatment.

P16 is a negative regulator of cellular proliferation, working through a negative feedback loop to downregulate cyclin-dependent kinases 4 and 6, and is encoded by the tumor suppressor gene cyclin-dependent kinase inhibitor 2A (12). In hrHPV infection, the oncoprotein E7 causes functional inactivation of the retinoblastoma gene product, resulting in the release of transcription factor E2F (13, 14). The downstream effect is the promotion of cell cycle progression and release of p16 gene from its transcriptional inhibition, causing an increase in p16 (15, 16).

Expression of the p16 protein correlates with the severity of dysplasia in cervical cells; therefore, p16 is a surrogate marker reflecting the oncogenic activity of hrHPV in cells (17–20). In a meta-analysis, only 2% of normal biopsies and 38% of CIN grade 1 cases showed diffuse staining for p16, compared with 68% for CIN grade 2 and 82% for CIN grade 3 (21). In HIV-negative women, p16 expression has high sensitivity (87%) for differentiating CIN grade 1 from CIN2/3 and lower specificity (76%), with histological diagnosis as the gold standard (19, 22). A similar study among women with HIV showed a sensitivity of 54% and specificity of 72% for detecting CIN2/3 (19, 22). It is not well understood why the sensitivity of p16 protein expression differs in the presence of HIV. P16 can be inactivated by mutations, deletions, loss of heterozygosity, or hypermethylation, resulting in reduced or negative expression in CIN (23).

Few studies have investigated the role of p16 in predicting CIN2+ recurrence after surgical treatment and the findings are equivocal (24–26). We evaluated the correlates of p16 expression in women with HIV and CIN2/3 at treatment with cryotherapy and the diagnostic utility of p16 immunohistochemistry in predicting recurrence of CIN2+ in the two-years following treatment.

## Materials and methods

### Participants and study procedures

This was a subgroup analysis of a randomized clinical trial conducted at the Coptic Hope Centre for Infectious Diseases in Nairobi, Kenya, from June 2011 to September 2016. The parent trial randomized 400 women with HIV and biopsy-confirmed CIN2/3 to cryotherapy or LEEP, as previously described (11). Briefly, eligible participants were randomized to cryotherapy or LEEP in a 1:1 allocation using a permuted-block randomization with a block size of 10. Investigators were masked to block number, size and sequence. Participants underwent Pap smear and confirmatory biopsy every 6-months for two-years to evaluate recurrence of CIN2+. This subgroup analysis includes previously reported epidemiologic and laboratory data (HIV and HPV genotyping) from this trial (7, 11). Any hrHPV infection was defined as any one of the following 14 genotypes at baseline cryotherapy treatment: 16, 18, 31, 33, 35, 39, 45, 51, 52, 56, 58, 59, 66, and 68. Infection with multiple hrHPV genotypes was defined as two or more hrHPV genotypes at baseline. Additional correlates investigated were age, marital status, age at sexual debut, education level (primary and below, secondary and above), number of lifetime sexual partners (1–2 vs. ≥3), CD4 cell count (<250, 250–499, ≥500 cells/mm^3^), plasma HIV viral load (detectable ≥ 60 copies/mL) vs. undetectable [<60 copies/mL], antiretroviral therapy (ART) duration (<2 years vs. ≥2 years), and CIN2/3 at cryotherapy. CIN2+ recurrence was defined as a consensus interpretation of CIN grade 2 or higher on cervical biopsy at any of the six-month intervals during the two-year follow-up period.

Ethical approvals were obtained from the Kenyatta National Hospital Ethics and Research Committee, University of Washington Institutional Review Board, US Centers for Disease Control and Prevention, and Aga Khan University Ethics Review Committee. Participants consent was waived as this study dealt with secondary data analysis.

### P16 immunohistochemistry

Immunohistochemistry (IHC) for p16 staining was performed and expression was assessed as previously described (24). In brief, 4 μm tissue sections were subjected to the following processes: deparaffinization, antigen retrieval with citrate at pH 9, endogenous peroxidase blocking, application of prediluted mouse anti-p16/INK4a (Medaysis, California, United States), and finally, application of horseradish peroxidase (HRP), specific 3,3′-diaminobenzidine (DAB), and counterstaining. Two independent pathologists reviewed the sections and reached a consensus. Positive p16 expression was defined as block staining when there was strong abnormal nuclear expression in a continuous segment of cells (at least 10–20 cells). Tissue sections of squamous cell carcinoma were used as the positive control.

### Statistical analysis

Baseline characteristics were compared using chi-square test for proportions. Binomial logistic regression was used to evaluate the correlates of p16 expression at baseline cryotherapy treatment. A multivariable model was constructed to assess covariates that were independently associated with p16 expression at baseline cryotherapy. The following covariates with p16 expression at *p* < 0.10 on univariate analysis were included in the multivariable model: any one hrHPV genotype, plasma HIV viral load, and ART duration. Number of lifetime sexual partners was excluded from the final multivariable model due to an unacceptably high number of missing data (23 of 160) that was likely to bias the results (27).

Cox proportional hazards regression models were used to derive hazard ratios (HR) and 95% confidence intervals (CI) for the association between p16 expression and CIN2+ recurrence during two-year follow-up. Follow-up time was calculated from the date of the initial cryotherapy to the first follow-up visit indicating a biopsy-confirmed diagnosis of CIN2+ recurrence. Women were censored at the date of the last Pap test if they were lost to follow-up, died, or had not experienced CIN2+ disease recurrence by two-years. Finally, logistic regression analysis was performed to determine the effect of the combination of p16 expression and hrHPV infection on CIN2+ recurrence.

## Results

Of the 200 women who were randomized to cryotherapy, 160 (80%) had a baseline cervical biopsy specimen available and were included in the analysis. Of the 40 women that were excluded, 33 had archival biopsy blocks that lacked adequate cervical tissue or lesions and seven had biopsy blocks that could not be conclusively identified. There were no differences in baseline demographics and clinical characteristics between women with and without archival biopsy blocks. The prevalence of p16 expression at the baseline cryotherapy treatment was 59% (*n* = 94), of which 40% (*n* = 38) were CIN2 and 60% (*n* = 56) were CIN3. The mean age was 37 years (standard deviation, 8 years) and 64% (*n* = 102) of women had secondary or higher education. The median age at sexual debut was 18 years [interquartile range (IQR), 16–20] and 55% (75/137) of women reported having had three or more lifetime sexual partners. The median antiretroviral therapy (ART) duration was 1.9 years (IQR, 0.5–4.7), median CD4 count was 374 cells/μL (IQR, 232–512), and 58% (*n* = 93) had undetectable HIV viral load (defined as <60 copies/mL). Higher proportion of women with three or more lifetime sexual partners (55.3% vs. 34.9%, *p* = 0.04), shorter ART duration (61.7% vs. 42.4%, *p* = 0.02), and detectable HIV viral load (48.9% vs. 31.8%, *p* = 0.03) had p16 positive biopsy results (Table 1).

**Table 1:** Baseline demographic and clinical characteristics by P16 status (*n* = 160).

Characteristics	*n* (%)		*p*-value^≠^
	p16 positive	p16 negative	
	(*n* = 94)	(*n* = 66)	
**Age, years**			
<35 years	38 (42.2)	27 (42.9)	0.94
≥35 years	52 (57.8)	36 (57.1)	
**Relationship status**			
Married/cohabiting	37 (41.1)	27 (42.2)	0.56
Divorced/widowed	24 (26.7)	21 (32.8)	
Single	29 (32.2)	16 (25.0)	
**Age at sexual debut**			
<18 years	33 (38.8)	24 (40.7)	0.82
≥18 years	52 (61.2)	35 (59.3)	
**Education**			
Primary and below	36 (38.3)	22 (33.3)	0.52
Secondary and above	58 (61.7)	44 (66.7)	
**Lifetime sexual partners** ^a^			
1–2	30 (31.9)	32 (48.5)	0.04
≥3	52 (55.3)	23 (34.9)	
Missing data	12 (12.9)	11 (16.7)	
**CD4 count (cells/mm** ^**3**^ **)**			
<250	31 (33.0)	15 (22.7)	0.32
250–499	40 (42.6)	30 (45.5)	
≥500	23 (24.5)	21 (31.8)	
**Duration of antiretroviral therapy**			
<2 years	58 (61.7)	28 (42.4)	0.02
≥2 years	36 (38.3)	38 (57.6)	
**Plasma HIV viral load** ^b^			
Undetectable (<60 copies/mL)	48 (51.1)	45 (68.2)	0.03
Detectable (≥60 copies/mL)	46 (48.9)	21 (31.8)	
**Biopsy result at cryotherapy**			
CIN grade 2	38 (40.4)	30 (45.5)	0.40
CIN grade 3 and higher	56 (59.6)	36 (54.5)	

^**≠**^Chi-square test. ^a^Missing data (*n* = 23). ^b^Minimum detection level < 60 copies/mL.

Overall, 145 (90.6%) of women with p16 biopsy results were positive for any one hrHPV genotype at baseline. Ninety-five percent (*n* = 89) of women with p16 positive biopsies had presence of any one hrHPV genotype compared to 85% (*n* = 56) of p16 negative biopsies (*p* = 0.04; Table 2). Singularly, only hrHPV genotype 58 was significantly associated with p16 expression (*p* = 0.01).

**Table 2:** Prevalence of high-risk HPV (hrHPV) genotypes by p16 expression at baseline treatment.

	P16 positive	P16 negative	*p*-value^≠^
	(*n* = 94)	(*n* = 66)	
Any hrHPV genotype^a^	89 (95%)	56 (85%)	0.04
Multiple hrHPV genotypes^b^	53 (56%)	34 (52%)	0.79
Type 16	24 (26%)	15 (23%)	0.68
Type 18	18 (19%)	13 (20%)	0.93
Type 31	7 (7%)	3 (5%)	0.46
Type 33	11 (12%)	6 (9%)	0.60
Type 35	22 (23%)	10 (15%)	0.20
Type 39	8 (9%)	5 (8%)	0.83
Type 45	7 (7%)	6 (9%)	0.71
Type 51	11 (12%)	5 (8%)	0.39
Type 52	19 (20%)	12 (18%)	0.80
Type 56	13 (14%)	8 (12%)	0.75
Type 58	29 (31%)	9 (14%)	0.01
Type 59	10 (11%)	7 (11%)	0.99
Type 66	10 (11%)	8 (12%)	0.77
Type 68	4 (4%)	5 (8%)	0.37

hrHPV, high-risk human papillomavirus. ^≠^Chi-square test. ^a^Defined as any one of the following hrHPV genotypes: 16, 18, 31, 33, 35, 39, 45, 51, 52, 56, 58, 59, 66, 68. ^b^Defined as two or more hrHPV genotypes.

In univariate logistic analysis, detectable HIV viral load (≥60 copies/mL), ≥3 lifetime sexual partners, and infection with any one hrHPV genotype was associated with increased odds of p16 expression at baseline (Table 3). Longer ART duration (≥2 years) was associated with 54% lower odds of p16 expression (OR = 0.46, 95% CI, 0.24–0.87) compared to less than 2 years of ART. In multivariable analysis, any one hrHPV genotype and ≥ 2 years of ART remained independently associated with higher and lower odds of p16 expression (OR = 3.7, 95% CI: 1.2–11.9; OR = 0.47, 95% CI: 20.24–40.9, respectively).

**Table 3:** Factors associated with positive p16 status at baseline cryotherapy treatment (n = 160).

	Univariate		Multivariate model^a^	
	OR (95% CI)	*p*-value	Adjusted OR (95% CI)	*p*-value
Any one hrHPV genotype^b^	3.17 (1.03–9.78)	0.04	3.73 (1.17–11.94)	0.026
Multiple hrHPV genotypes^c^	1.11 (0.57–2.12)	0.79	NA	NA
**CD4 cell count, cells/mm** ^**3**^				
<250	1.0 (Reference)	NA	NA	NA
250–499	0.71 (0.32–1.55)	0.39	NA	NA
≥500	0.53 (0.23–1.25)	0.15	NA	NA
Detectable plasma HIV viral load^d^	1.43 (1.01–2.03)	0.03	1.96 (0.99–3.90)	0.054
**Antiretroviral therapy**				
<2 years	1.0 (Reference)	NA	1.0 (Reference)	NA
≥2 years	0.46 (0.24–0.87)	0.02	0.47 (0.24–0.91)	0.026
Baseline CIN3 (ref: CIN2)	1.22 (0.62–2.38)	0.57	NA	NA
≥3 lifetime sex partners (ref: <3)	1.62 (1.03–2.54)	0.04	NA	NA

CIN2, cervical intraepithelial neoplasia grade 2; CIN3, cervical intraepithelial neoplasia grade 3; hrHPV, high-risk human papillomavirus; OR, odds ratio.^a^Multivariable model included ART duration, plasma HIV viral load and infection with any one hrHPV genotype.^b^Defined as any one of the following hrHPV genotypes: 16, 18, 31, 33, 35, 39, 45, 51, 52, 56, 58, 59, 66, 68.^c^Defined as two or more hrHPV genotypes.^d^Minimum detection level < 60 copies/mL.

**Figure 1:**
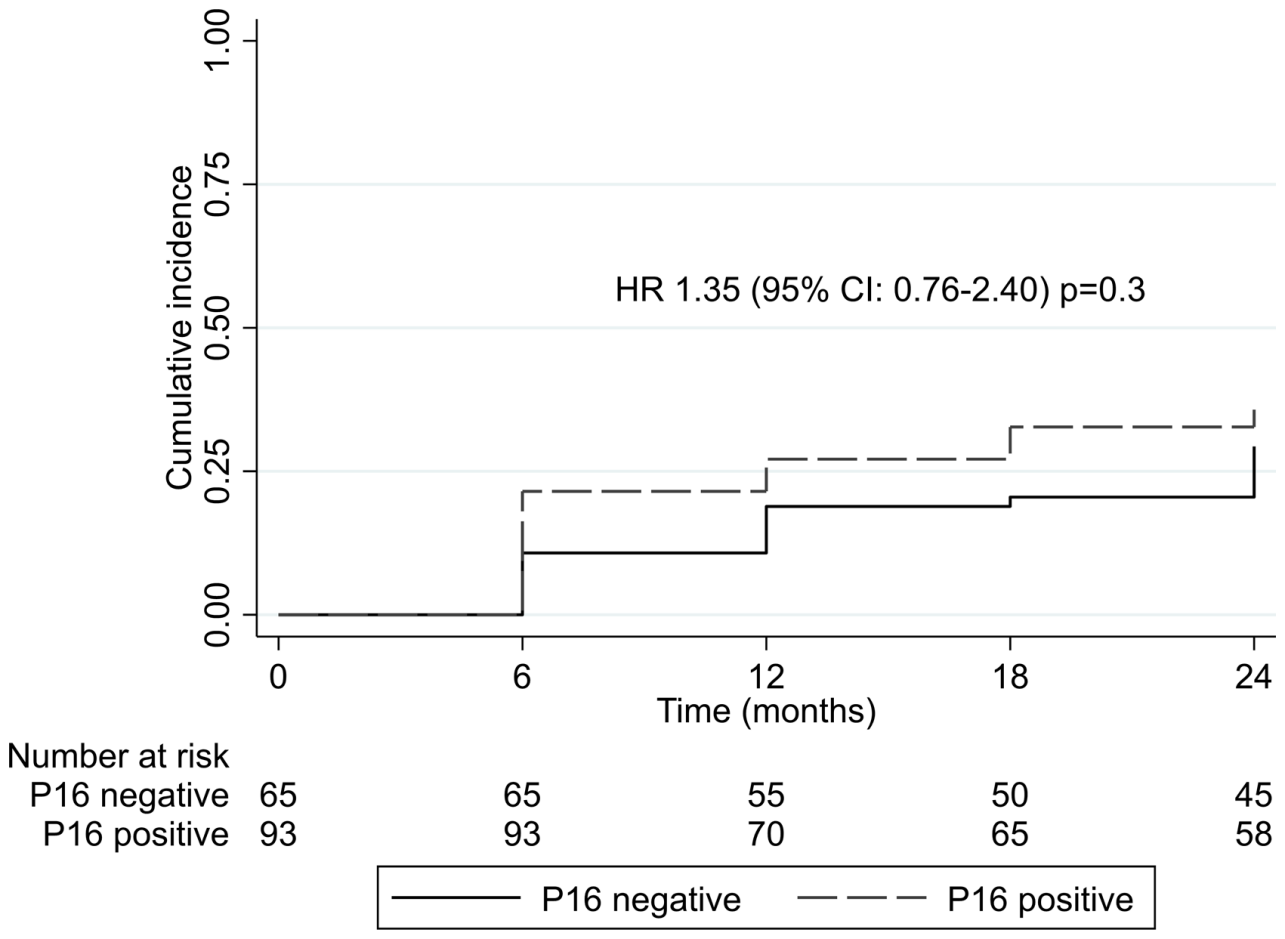
Kaplan–Meier cumulative incidence by p16 expression at baseline. CI, confidence interval; HR, hazard ratio.

During two-year follow-up, 32% (*n* = 51) of women and CIN2+ treated with cryotherapy at baseline had CIN2+ recurrence. Of these, 65% (*n* = 33) were positive for p16 expression at baseline. Of the 94 women who were p16 positive at baseline, 35% (*n* = 33) had CIN2+ recurrence. The two-year rate of CIN2+ recurrence among women with p16 expression was 22 per 100 person-years compared to 16 per 100 person-years among women without p16 expression at baseline (*p* = 0.14). CIN2+ recurrence risk did not significantly differ by p16 expression at baseline (HR = 1.35; 95% CI, 0.76–2.40, *p* = 0.31; Figure 1). Combining p16 expression with the presence of any one hrHPV genotype had no additional benefit in predicting CIN2+ recurrence (HR = 1.38; 95% CI, 0.78–2.43, *p* = 0.27; Table 4). There were no differences in CIN2+ recurrence by CIN2/3 at baseline cryotherapy (35% among women with CIN2 and 34% among those with CIN3).

**Table 4:** Risk of cervical disease recurrence (CIN2+) by combined p16 expression and any one hrHPV genotype and simultaneous multiple hrHPV genotypes at baseline cryotherapy treatment.

p16 and hrHPV status	Hazard ratio (95% CI)	*p*-value
p16 negative, any one hrHPV negative	0.84 (0.26–2.70)	0.77
p16 negative, any one hrHPV positive	0.73 (0.40–1.32)	0.28
p16 positive, any one hrHPV negative	1.19 (0.29–4.89)	0.81
p16 positive, any one hrHPV positive	1.38 (0.78–2.43)	0.27
p16 negative, multiple hrHPV negative	0.86 (0.38–1.91)	0.71
p16 negative, multiple hrHPV positive	0.67 (0.31–1.43)	0.30
p16 positive, multiple hrHPV negative	0.78 (0.39–1.57)	0.49
p16 positive, multiple hrHPV positive	1.64 (0.93–2.89)	0.09

CIN2+, cervical intraepithelial neoplasia grade 2 or higher; CI, confidence interval; hrHPV, high-risk human papillomavirus.

## Discussion

In this study of women with HIV and CIN2+, prevalence of p16 expression at initial cryotherapy treatment was 59%. Infection with any one hrHPV genotype and longer duration (≥2 years) of ART were independently associated with p16 expression even after controlling for HIV viral suppression. In our population of women with HIV, baseline p16 expression was not associated with recurrent CIN2+ disease in the two-years following cryotherapy. Addition of p16 expression to hrHPV testing did not increase the predictive value of CIN2+ recurrence among women with HIV.

The role of p16 as a predictor of recurrent cervical disease is inconclusive, with limited and contradictory results. A large study in China reported a significant association between p16 and recurrent CIN2+ disease after conization and two-year follow-up (25). Compared with our study, the women were on average 10 years older and both their HIV and hrHPV status at baseline treatment were not disclosed, thus we cannot directly compare our findings. In a smaller study of HIV-uninfected women with CIN2+, cervical disease recurrence was 37% at 18 months and the positive predictive value of p16 for recurrent CIN2+ disease was 42% (24). Similarly, in a study in Mexico, there was no association between the CIN2+ recurrence-free period and p16 expression (*p* = 0.57) and few women were living with HIV (5%) (26). Moreover, p16 expression has been shown to be less sensitive than insulin-like growth factor II mRNA-binding protein (IMP3) expression at predicting progression and recurrence of HSIL among women living with HIV (28).

P16 in combination with Ki-67 (dual stain) may have a role in the triage of cervical lesions during screening. In women testing hrHPV positive, where most hrHPV infections resolve spontaneously, a negative p16/Ki-67 dual-stain result is used to inform an extended follow-up interval for 3 years while a p16/Ki-67 positive result indicates the need for repeat testing, colposcopy, or immediate treatment based on the risk threshold (29). P16 has been shown to add value in the triage of women with atypical squamous cells of undetermined significance (ASCUS), with a positive p16 result increasing the specificity for a high-grade cervical disease diagnosis compared to HPV DNA alone (30). Among women living with HIV, p16 expression has shown decreased sensitivity and increased specificity for CIN2+ diagnosis when combined with other screening modalities (22).

In our study, p16 expression was associated with any one hrHPV genotype and individually, hrHPV genotype 58, but not with hrHPV types 16 or 18, singly or combined. While women with HIV are more likely to have persistent and multiple hrHPV infections compared to their HIV-uninfected peers (31–33), longer duration of ART and viral suppression have been shown to be associated with lower hrHPV prevalence in women with HIV (34). This is in agreement with our findings in which longer ART duration was inversely associated with p16 expression even after adjusting for viral suppression and hrHPV infection. Similarly, detectable HIV viral load was associated with higher odds of p16 expression, although this was borderline-significant (*p* = 0.054) in the adjusted model. Recurrence of cervical disease was not associated with p16 expression and combining p16 with any one or multiple hrHPV genotypes had no additional benefit for risk prediction in our population of women with HIV and CIN2/3.

Our study had several limitations. These were archival blocks with no record of the quality of fixation used, which may have affected p16 staining, leading to spurious loss of expression. We did not stain for p16 in later biopsies of recurrent CIN2+ disease and thus, are not able to establish a temporal relationship between p16 expression and cervical disease recurrence. In addition, 20% (*n* = 40) women who had cryotherapy in parent study were excluded for various reasons. The small sample size may have limited our ability to detect all correlates of p16 expression and their association with CIN2+ recurrence. Finally, we did not have information on lesion size, which may have been helpful to further explain our findings.

In conclusion, p16 expression was uncommon among women with HIV with CIN2+. P16 expression was associated with any one hrHPV genotype, shorter ART duration, and detectable HIV viral load. P16 expression alone or in combination with hrHPV infection was not useful for predicting recurrent cervical disease after cryotherapy in this population.

## Data availability statement

The original contributions presented in the study are included in the article/supplementary material, further inquiries can be directed to the corresponding author.

## Ethics statement

The studies involving humans were approved by Aga Khan University Scientific and Ethics Review Committee. The studies were conducted in accordance with the local legislation and institutional requirements. The ethics committee/institutional review board waived the requirement of written informed consent for participation from the participants or the participants’ legal guardians/next of kin because primary research work was on archived laboratory materials and secondary data analysis of parent study.

## Author contributions

DM: Conceptualization, Data curation, Formal Analysis, Investigation, Methodology, Writing – original draft, Writing – review & editing. MC: Conceptualization, Funding acquisition, Supervision, Writing – review & editing. MT: Funding acquisition, Supervision, Writing – review & editing. ZM: Methodology, Resources, Writing – review & editing. JW: Investigation, Writing – review & editing. SG: Writing – review & editing. EU: Resources, Writing – review & editing. NM: Writing – review & editing. SaS: Resources, Writing – review & editing. ShS: Investigation, Resources, Writing – review & editing. CM: Conceptualization, Supervision, Writing – original draft, Writing – review & editing.

## References

[1] Sung H Ferlay J Siegel RL Laversanne M Soerjomataram I Jemal A. Global cancer statistics 2020: GLOBOCAN estimates of incidence and mortality worldwide for 36 cancers in 185 countries. CA Cancer J Clin. (2021) 71:209–49. doi: 10.3322/caac.21660, PMID: 33538338

[2] Ngcobo N Jaca A Iwu-Jaja CJ Mavundza E. Reflection: burden of cervical cancer in sub-Saharan Africa and progress with HPV vaccination. Curr Opin Immunol. (2021) 71:21–6. doi: 10.1016/j.coi.2021.03.006, PMID: 33857884

[3] Sweet K Bosire C Sanusi B Sherrod CJ Kwatampora J Waweru W. Prevalence, incidence, and distribution of human papillomavirus types in female sex workers in Kenya. Int J STD AIDS. (2020) 31:109–18. doi: 10.1177/0956462419884454, PMID: 31948341 PMC7031817

[4] de Vuyst H Mugo N Chung M McKenzie K Nyongesa-Malava E Tenet V. Prevalence and determinants of human papillomavirus infection and cervical lesions in HIV-positive women in Kenya. Br J Cancer. (2012) 107:1624–30. doi: 10.1038/bjc.2012.441, PMID: 23033006 PMC3493776

[5] Nicol AF Fernandes ATG Bonecini-Almeida MG. Immune response in cervical dysplasia induced by human papillomavirus: the influence of human immunodeficiency virus-1 co-infection-review. Mem Inst Oswaldo Cruz. (2005) 100:1–12. doi: 10.1590/S0074-02762005000100001, PMID: 15867955

[6] Doorbar J. Latent papillomavirus infections and their regulation. Curr Opin Virol. (2013) 3:416–21. doi: 10.1016/j.coviro.2013.06.003, PMID: 23816390

[7] Chung MH de Vuyst H Greene SA Mugo NR Querec TD Nyongesa-Malava E. Human papillomavirus persistence and association with recurrent cervical intraepithelial neoplasia after cryotherapy vs loop electrosurgical excision procedure among HIV-positive women: a secondary analysis of a randomized clinical trial. JAMA Oncol. (2021) 7:1514–20. doi: 10.1001/jamaoncol.2021.2683, PMID: 34351377 PMC8343498

[8] Maza M Schocken CM Bergman KL Randall TC Cremer ML. Cervical precancer treatment in low-and middle-income countries: a technology overview. J Glob Oncol. (2017) 3:400–8. doi: 10.1200/JGO.2016.003731, PMID: 28831448 PMC5560450

[9] Greene SA McGrath CJ Lehman DA Marson KG Trinh TT Yatich N. Increased cervical human immunodeficiency virus (HIV) RNA shedding among HIV-infected women randomized to loop electrosurgical excision procedure compared to cryotherapy for cervical intraepithelial neoplasia 2/3. Clin Infect Dis. (2018) 66:1778–84. doi: 10.1093/cid/cix1096, PMID: 29272368 PMC6248794

[10] Debeaudrap P Sobngwi J Tebeu P-M Clifford GM. Residual or recurrent precancerous lesions after treatment of cervical lesions in human immunodeficiency virus–infected women: a systematic review and meta-analysis of treatment failure. Clin Infect Dis. (2019) 69:1555–65. doi: 10.1093/cid/ciy1123, PMID: 30602038 PMC6792085

[11] Greene SA De Vuyst H John-Stewart GC Richardson BA McGrath CJ Marson KG. Effect of cryotherapy vs loop electrosurgical excision procedure on cervical disease recurrence among women with HIV and high-grade cervical lesions in Kenya: a randomized clinical trial. JAMA. (2019) 322:1570–9. doi: 10.1001/jama.2019.14969, PMID: 31638680 PMC6806442

[12] Sherr CJ Roberts JM. CDK inhibitors: positive and negative regulators of G1-phase progression. Genes Dev. (1999) 13:1501–12. doi: 10.1101/gad.13.12.1501, PMID: 10385618

[13] Jabbar SF Abrams L Glick A Lambert PF. Persistence of high-grade cervical dysplasia and cervical cancer requires the continuous expression of the human papillomavirus type 16 E7 oncogene. Cancer Res. (2009) 69:4407–14. doi: 10.1158/0008-5472.CAN-09-0023, PMID: 19435895 PMC3006677

[14] Yamato K Yamada T Kizaki M Ui-Tei K Natori Y Fujino M. New highly potent and specific E6 and E7 siRNAs for treatment of HPV16 positive cervical cancer. Cancer Gene Ther. (2008) 15:140–53. doi: 10.1038/sj.cgt.7701118, PMID: 18157144

[15] Khleif SN DeGregori J Yee CL Otterson GA Kaye FJ Nevins JR. Inhibition of cyclin D-CDK4/CDK6 activity is associated with an E2F-mediated induction of cyclin kinase inhibitor activity. Proc Natl Acad Sci. (1996) 93:4350–4. doi: 10.1073/pnas.93.9.4350, PMID: 8633069 PMC39540

[16] Münger K Werness B Dyson N Phelps W Harlow E Howley P. Complex formation of human papillomavirus E7 proteins with the retinoblastoma tumor suppressor gene product. EMBO J. (1989) 8:4099–105. doi: 10.1002/j.1460-2075.1989.tb08594.x, PMID: 2556261 PMC401588

[17] Kishore V Patil AG. Expression of p16INK4A protein in cervical intraepithelial neoplasia and invasive carcinoma of uterine cervix. J Clin Diagn Res. (2017) 11:EC17. doi: 10.7860/JCDR/2017/29394.10644 PMC5713738 29207716

[18] Lin J Albers AE Qin J Kaufmann AM. Prognostic significance of overexpressed p16INK4a in patients with cervical cancer: a meta-analysis. PLoS One. (2014) 9:e106384. doi: 10.1371/journal.pone.0106384, PMID: 25188353 PMC4154680

[19] Xing Y Wang C Wu J. Expression of geminin, p16, and Ki67 in cervical intraepithelial neoplasm and normal tissues. Medicine. (2017) 96:e7302. doi: 10.1097/MD.0000000000007302, PMID: 28658133 PMC5500055

[20] Shi Q Xu L Yang R Meng Y Qiu L. Ki-67 and P16 proteins in cervical cancer and precancerous lesions of young women and the diagnostic value for cervical cancer and precancerous lesions. Oncol Lett. (2019) 18:1351–5. doi: 10.3892/ol.2019.10430, PMID: 31423197 PMC6607340

[21] Tsoumpou I Arbyn M Kyrgiou M Wentzensen N Koliopoulos G Martin-Hirsch P. p16INK4a immunostaining in cytological and histological specimens from the uterine cervix: a systematic review and meta-analysis. Cancer Treat Rev. (2009) 35:210–20. doi: 10.1016/j.ctrv.2008.10.005, PMID: 19261387 PMC2784486

[22] McGrath CJ Garcia R Trinh TT Richardson BA John-Stewart GC Nyongesa-Malava E. Role of p16 testing in cervical cancer screening among HIV-infected women. PLoS One. (2017) 12:e0185597. doi: 10.1371/journal.pone.0185597, PMID: 29023464 PMC5638250

[23] Gonçalves JE Andrade CV Russomano FB Nuovo GJ Amaro-Filho SM Carvalho MO. The role of p16 as putative biomarker for cervical neoplasia: a controversial issue? MedicalExpr. (2017) 4:4. doi: 10.5935/MedicalExpress.2017.06.01

[24] Fonseca FV Tomasich FDS Jung JE Maestri CA Carvalho NS. The role of P16 ink4a and P53 immunostaining in predicting recurrence of HG-CIN after conization treatment. Rev Col Bras Cir. (2016) 43:35–41. doi: 10.1590/0100-69912016001008, PMID: 27096855

[25] Wang X Zhao Y Zou X Wang L. Relationship of P16 and Ki67 in recurrence of HPV infection and cervical intraepithelial neoplasia. Int J Clin Exp Pathol. (2020) 13:3174. PMID: 33425118 PMC7791391

[26] Arredondo-Gálvez CG Acuña-González D Cantú-de-León D Chanona-Vilchis JG Avilés-Salas A González-Enciso A. Association of p16 and Ki-67 with risk of recurrence in previously treated cervical high-grade squamous intraepithelial lesions. Gynecol Obstet Investig. (2021) 86:293–8. doi: 10.1159/000515894, PMID: 34111875

[27] Scheffer J. (2002). Dealing with missing data, Research Letters in the Information and Mathematical Sciences, (2002) 3:153–160.

[28] del Gobbo A Bonoldi E Cribiù FM Franceschetti I Matinato C Fiori S. Insulin-like growth factor II mRNA binding protein 3 (IMP3) expression in cervical intraepithelial neoplasia and its relationship with HIV-infection status. Sex Health. (2015) 12:22–6. doi: 10.1071/SH13144 25427240

[29] Clarke MA Cheung LC Castle PE Schiffman M Tokugawa D Poitras N. Five-year risk of cervical precancer following p16/Ki-67 dual-stain triage of HPV-positive women. JAMA Oncol. (2019) 5:181–6. doi: 10.1001/jamaoncol.2018.4270, PMID: 30325982 PMC6439556

[30] Zhu Y Ren C Yang L Zhang X Liu L Wang Z. Performance of p16/Ki67 immunostaining, HPV E6/E7 mRNA testing, and HPV DNA assay to detect high-grade cervical dysplasia in women with ASCUS. BMC Cancer. (2019) 19:1–9. doi: 10.1186/s12885-019-5492-9 30917784 PMC6437959

[31] Swai P Rasch V Linde DS Mchome B Manongi R Wu CS. Persistence and risk factors of high-risk human papillomavirus infection among HIV positive and HIV negative tanzanian women: a cohort study. Infect Agents Cancer. (2022) 17:1–11. doi: 10.1186/s13027-022-00442-2 PMC9188099 35690838

[32] Thorsteinsson K Ladelund S Storgaard M Katzenstein TL Johansen IS Pedersen G. Persistence of cervical high-risk human papillomavirus in women living with HIV in Denmark–the SHADE. BMC Infect Dis. (2019) 19:1–10. doi: 10.1186/s12879-019-4377-5 31438877 PMC6706931

[33] Liu G Sharma M Tan N Barnabas RV. HIV-positive women have higher risk of human papilloma virus infection, precancerous lesions, and cervical cancer. AIDS. (2018) 32:795–808. doi: 10.1097/QAD.0000000000001765, PMID: 29369827 PMC5854529

[34] Kelly H Chikandiwa A Vilches LA Palefsky JM de Sanjose S Mayaud P. Association of antiretroviral therapy with anal high-risk human papillomavirus, anal intraepithelial neoplasia, and anal cancer in people living with HIV: a systematic review and meta-analysis. Lancet HIV. (2020) 7:e262–78. doi: 10.1016/S2352-3018(19)30434-5, PMID: 32109408

